# Exploring the impact of platform leadership on employee innovative behavior: a sequential explanatory mixed method

**DOI:** 10.3389/fpsyg.2025.1435683

**Published:** 2025-05-21

**Authors:** Mengting Yuyi, Ying Li, Xin Zhu, Muhammad Shahid Khan

**Affiliations:** ^1^College of Foreign Languages, Fujian Normal University, Fuzhou, China; ^2^School of Foreign Studies, Guangxi University of Science and Technology, Liu Zhou, China; ^3^Development Planning Department, Guangxi University of Science and Technology, Liu Zhou, China; ^4^College of Innovation and Management, Suan Sunandha Rajabhat University, Bangkok, Thailand

**Keywords:** platform leadership, organizational learning, knowledge sharing, coworker support, psychological empowerment, employee innovative behavior

## Abstract

This study uses the Ability—Motivation—Opportunity framework to explore the influence mechanism of platform leadership on employees’ innovation behavior. This study examines organizational learning, knowledge sharing, coworker support, and psychological empowerment as mediating variables, employee innovation behavior as the dependent variable. This study constructs a multi-mediated model to analyze the impact of platform leadership on employee innovative behavior, which includes 13 hypotheses. The study primarily focuses on data collected from China’s Shanxi and Guangxi regions, encompassing manufacturing, software and information services, internet companies, and enterprises establishing digital platforms, with individual employees as the subjects of investigation. This research employed a mixed-method approach, following a standardized research process in a sequential explained process. Research one involved the analysis of questionnaires from 518 employees, which applies to a structural equation model. The model’s multiple mediation effects are confirmed using the bootstrap method. Research two comprised semi-structured interviews with 24 enterprise employees. Text data mining is conducted using Python and the open-source Chinese tool “Weiciyun” to explore the underlying mechanisms between platform leadership and innovation behavior. This study underscores the practical contributions of platform leadership to organizational development by demonstrating its positive impact on organizational learning, knowledge sharing, coworker support, and psychological empowerment, all of which significantly enhance employee innovation behavior. Organizational learning emerges as the most critical mediator. The findings provide actionable insights for leadership policies, emphasizing the importance of cultivating a learning-oriented culture, fostering knowledge sharing, and empowering employees psychologically. By implementing these strategies, leaders can effectively drive innovation and improve organizational performance, offering a clear pathway for leadership practices to enhance adaptability and innovation within organizations.

## 1 Introduction

The digital transformation of the economy has spurred the growth of platform—based economies, characterized by data—driven operations, platform support, and networked collaboration. To meet personalized market demands, enterprises are increasingly adopting flatter, decentralized, and platform—oriented organizational structures (Xie, 2022). In 2014, scholar Hao Xuguang introduced the concept of “platform leadership,” a novel model combining platform thinking and leadership practices. This leadership involves leaders creating internal platforms to empower employees, fostering mutual growth and a dynamic, spiraling synergy between the organization and its employees.

In today’s volatile market, innovation is critical for survival and competitive advantage, making employee innovation behavior a key focus. Recent studies indicate that platform leadership positively impacts various forms of employee innovation, including proactive, deviant, and breakthrough innovation behaviors (Ma et al., 2024; Yang and Xiao, 2024; Min and Kim, 2021). However, research on the mechanisms through which platform leadership influences employee innovation remains limited, with issues such as inconsistent definitions, narrow perspectives, and methodological constraints. The exploration of mediating variables is particularly scarce, and the internal dynamics of platform leadership’s impact on innovation behaviors are yet to be fully understood (Xu, 2022; Zhou et al., 2023). Further research is needed to address these gaps and deepen our understanding of this emerging leadership paradigm.

This study aims to explore the factors influencing employees’ performance behaviors, and its research framework is based on the AMO model of performance. This model was proposed by Vroom in the framework of the expectancy theory in 1964 and later refined by Blumberg. It posits that employees’ performance behaviors are comprehensively influenced by ability, motivation, and opportunity, and it is an important theoretical framework from the “behavioral perspective” (Vroom, 1964; Pringle and Blumberg, 1986). In this study, psychological empowerment is classified as an ability factor. It enhances employees’ confidence and self-efficacy when performing innovative tasks, thus laying a foundation for innovation. Knowledge sharing serves as a motivation factor. When employees share knowledge, they can be exposed to new ideas, form a positive feedback loop, and strengthen their motivation for innovation. Platform leadership, organizational learning, and coworker support are considered opportunity factors. Platform leaders create an innovative culture and build platforms for resource sharing. Organizational learning disseminates new knowledge and technologies, and coworker support alleviates psychological pressure. Together, these three factors provide employees with opportunities for innovation and increase the likelihood of innovation. Therefore, the AMO theory is suitable for exploring the relationships between these factors and the innovative behaviors of employees in platform enterprises.

This study employs a sequential explanatory design to analyze the internal mechanisms through which platform leadership influences employee innovation behavior. This design aims to contrast and interpret the findings of quantitative analysis with qualitative data. Initially, the research framework is established through quantitative data, followed by the collection of qualitative data to aid in explaining the results of the quantitative analysis. Two reasons justify the use of a mixed-methods approach: first, the variables and model in this study are synthesized from existing literature, known variables, and current theories. Following Remenyi et al. (1998), who suggest quantitative research for such purposes, this study begins with a quantitative approach. Second, platform leadership is a novel concept rooted in Chinese context, with many uncertainties and unknown internal mechanisms requiring in-depth and open-ended exploration. Using only quantitative or qualitative methods alone cannot capture descriptive details of the internal mechanisms, nor can they fully describe the developmental trends of the issue. Therefore, combining both quantitative and qualitative data is essential for a comprehensive analysis of the internal mechanisms through which platform leadership influences employee innovation behavior.

In summary, this study, based on the ability-motivation-opportunity theory, introduces organizational learning, knowledge sharing, coworker support, and psychological empowerment as mediating variables to construct an integrated, multiple-mediator model of platform leadership’s impact on employee innovation behavior. The study has three primary objectives: first, to investigate whether platform leadership influences employee innovation behavior; second, to explore the mechanisms of this influence; and third, to examine whether organizational learning, knowledge sharing, psychological empowerment, and coworker support serve as mediators in this process.

## 2 Literature review and hypotheses

### 2.1 Platform leadership and employee innovative behavior

Platform leadership, emphasizes equality and sharing in a dynamic organizational environment (Zhao, 2018). This leadership style encourages resource sharing and common goal development, activating potential and enhancing motivation (Wang et al., 2022). It is characterized by mutual fulfillment and growth. The theoretical foundation of platform leadership is rooted in the third wave of Maslow’s humanistic psychology. Hao (2014) posits that individuals have the potential for self-actualization, and leaders should provide a platform for this. Platform leadership differs from traditional styles by fostering a collaborative, interactive, and growth-oriented environment. Transform Charismatic leadership relies on personal influence and top-down motivation, neglecting employee development (Xu, 2022). Service leadership emphasizes altruistic service but is one-sided and less interactive (Russell, 2001; Lu et al., 2021). Authoritative leadership focuses on top-down empowerment for performance, whereas platform leadership promotes mutual influence and growth. Inclusive leadership creates a supportive environment but overlooks leader development (Xiong et al., 2024). Platform leadership integrates elements from these styles, emphasizing mutual growth, equal interaction, and continuous learning (Yang and Zhang, 2023).

Platform leadership effectively stimulates employees’ innovative behavior as an opportunity factor by creating an environment, building platforms, and providing resources (Hao, 2014). Firstly, leaders with inclusive traits create a relaxed environment for employees’ innovation (Li, 2019). They accept the diverse contributions and differences of employees and tolerate mistakes (He, 2020). This inclusiveness gives employees a sense of psychological security, so they don’t have to worry about the negative consequences of innovation failure (Zhou and Cheng, 2018). As a result, they are more willing to participate in creative activities and gain innovation opportunities (Cropanzano and Mitchell, 2005; Javed et al., 2018). Secondly, leaders capable of building and optimizing growth platforms are crucial. In an era when employees aspire to realize their self—worth, platform—based leaders pay attention to employees’ needs and build platforms for them, enabling employees to unleash their creativity and tap into their innovation potential (Chen, 2016). Meanwhile, the created innovative atmosphere also promotes the generation and application of innovative ideas, providing employees with opportunities to put their innovative ideas into practice. Moreover, leaders who encourage change provide resource support for employees’ innovation (Li et al., 2019), ensuring the implementation of employees’ innovation opportunities at the practical level and enabling employees to carry out innovation activities with resource support (Hao et al., 2021; Fan et al., 2021).

*H1*: Platform leadership significantly positively influences employee innovative behavior.

### 2.2 Hypothetical development of organizational learning

Most scholars have affirmed the influence of leadership behavior on organizational learning (Shu, 2018; Ahsan, 2021; He, 2020; Wei, 2020). Aragón-Correa (2007) views it as knowledge acquisition and sharing to elevate internal knowledge levels. Platform leadership emphasizes mutual growth with subordinates. Leaders learn continuously and share knowledge with employees, promoting their learning and knowledge—sharing (Hao et al., 2021). This helps create a learning culture, facilitating organizational learning. Moreover, it plans long—term for the enterprise, integrates employee and enterprise development, encourages decision—making participation, and shares a vision (Hao, 2014; Yang X. et al., 2022). Hence, this study proposes the following hypothesis:

*H2*: Platform leadership significantly positively influences organizational learning.

Organizations and individuals can enhance competitiveness through learning driven innovation (Schon, 1997). Studies show organizational learning positively impacts employee innovative behavior and performance (Zhao and Wang, 2021; Han, 2017). As enterprises merge with digital economies, learning is crucial. This article posits organizational learning has three dimensions: learning commitment, vision sharing, and open—mindedness. Strong organizational learning will encourage employees within the organization to learn from each other, stimulate their learning interest, and promote the emergence of their innovation awareness (Cohen and Caner, 2016). On the contrary, weak organizational learning will lead to employees’ lack of learning interest and ability, insufficient communication and interaction, and a tendency to choose conservative behaviors. Thus, the following hypothesis is proposed:

*H6*: Organizational learning significantly positively impacts employees’ innovative behavior.

Leadership style is vital as it guides employees’ behavior (Wang and Chen, 2012). Scholars suggest organizational learning mediates between leadership and performance (Wang, 2021; Slater and Narver, 1995). Higher organizational—learning perception promotes knowledge—sharing and boosts performance (Lv, 2013). Platform leaders can enhance team learning and innovation (Li et al., 2022; Xu, 2022). Combining Hypothesis 2 and Hypothesis 6, this study proposes that platform leadership will affect the level of organizational learning, and the level of organizational learning will stimulate employees’ innovative behavior. Therefore, the following hypothesis is proposed:

*H10*: Organizational learning mediates the relationship between platform leadership and employee innovative behavior.

### 2.3 Hypothetical development of knowledge sharing

According to the AMO theory, as an opportunity factor, platform—type leaders influence employees’ knowledge sharing through the following approaches. Leaders create opportunities for knowledge exchange and promote knowledge transfer by building online knowledge management platforms, organizing cross—departmental projects, and holding sharing sessions (Appelbaum et al., 2000). Meanwhile, they construct an ecological environment for the career platform, align the organizational goals with employees’ goals, create a favorable environment, enhance employees’ intrinsic motivation to share knowledge, and eliminate their concerns (Yang and Xu, 2016; Russell and Marie, 2005). They also meet employees’ needs through interaction and weaken employees’ perception of loss. In this way, it can improve employees’ willingness and ability to share knowledge, and promote knowledge sharing and innovation within the organization (Xu, 2022; Wang and Hao, 2023). Therefore, the following hypothesis is proposed:

*H3*: Platform leadership exerts a significant positive influence on knowledge sharing.

Antonacopoulou and Gabriel (2001) emphasized the importance of knowledge for innovation. They posited that an organization’s knowledge assets are directly proportional to its innovation level, and the creative process follows the knowledge spiral. The core of knowledge management is to promote the dissemination of knowledge sharing (Jielin et al., 2020). The employees’ willingness to share knowledge determines the knowledge—sharing behavior within the organization (Christopher and Ken, 2006). Xu (2022) found that the willingness to share knowledge mediates the relationship between platform—type leadership and employees’ innovative behavior. Chen et al. (2018) pointed out that internal knowledge sharing is the key to improving individual innovation ability. Knowledge sharing can promote communication, cooperation, reduce conflicts, and foster the emergence of creativity (Yang et al., 2017). Numerous studies support the positive impact of knowledge sharing on employees’ innovative behavior (Ye, 2021; Tang Y., 2021; Zhang et al., 2014). Therefore, the following hypothesis is proposed:

*H7*: Knowledge sharing significantly enhances employee innovative behavior.

Platform leaders create and develop a shared vision with employees, redirecting employees’ focus from self-interest to the overall interests of the organization, thereby enhancing the willingness of team members to cooperate. In an organizational culture that is unanimously recognized by all employees, employees are more proactive. When their self-actualization needs are met, they actively communicate to acquire experiences and skills beyond the existing knowledge and explore diverse solution paths, which helps both individuals and the organization achieve innovation goals (Xin et al., 2020; Hao et al., 2021). Meanwhile, knowledge sharing, as a crucial part, reduces employees’ sense of uncertainty during the creative process (Mittal and Dhar, 2015), enabling them to be more innovative. In recent years, knowledge sharing is often regarded as a mediating variable in the impact of other factors on employees’ innovative behavior (Rao Jada et al., 2019). Du et al. (2018) found that social interaction can encourage employees’ innovative behavior by influencing the degree of knowledge sharing in the organization. Studies by Zhou and Cheng (2018) confirmed the mediating role of knowledge sharing in the impact of shared leadership on employees’ innovative behavior. Ma (2020) demonstrated that knowledge sharing plays a mediating role between the diverse work atmosphere and the innovative behavior of knowledge-based employees. Therefore, the following hypothesis is proposed:

*H11*: Knowledge sharing mediates the relationship between platform leadership and employee innovative behavior.

### 2.4 Hypothetical development of coworker supported

Platform leaders emphasize the shared career platform, help achieve common goals, and are good at establishing a collaborative and inclusive culture (Zhang, 2022; Tang R., 2021). Research has found that leadership behaviors focusing on emotional intelligence and interpersonal skills can create an inclusive and empathetic culture in the organization, which helps establish a supportive work environment, increases peer support, and enhances employees’ well-being and work engagement (Xue and Zhao, 2016). The study by Yang and Li (2013) shows that the level of coworker support is influenced by ethical leadership. Positive information such as relational help and normative, appropriate behaviors demonstrated by leaders can promote mutual trust and support among employees, creating a positive atmosphere and raising the level of coworker support (Ding, 2019). Their research also found that leaders’ inclusive traits contribute to building an inclusive culture and a supportive work environment, increasing communication, help, and support among colleagues. Platform leaders positively impact coworker support by creating a collaborative, supportive, and inclusive work environment that promotes trust, communication, and interdependence. Therefore, the following hypothesis is proposed:

*H4*: Platform leadership positively influences coworker support.

According to the AMO theory, coworker support, as an opportunity factor, influences employees’ innovative behavior. In the organization, coworker support provides opportunities for information exchange and knowledge sharing. Coworker instrumental support can make up for employees’ lack of knowledge and skills, facilitating the implementation of innovation tasks (Yan, 2019; Shi, 2020). Moreover, coworker support can create a favorable innovation atmosphere and communication opportunities. Emotional support can relieve psychological fatigue, strengthen motivation, and stimulate willingness (Deng et al., 2020). Given the risk of innovation, coworker emotional support and responsibility—sharing can buffer negative impacts, enabling employees to have the courage to continue innovating and increasing the likelihood of innovation success (Subrahmaniam and Rangaraj, 2008; Schumpeter, 1934). Existing studies have found that coworker support has a positive impact on employees’ innovative behavior and creativity (Zakarya, 2019; Deng et al., 2020). Therefore, the following hypothesis is proposed:

*H8*: Coworker support positively affects employee innovative behavior.

In summary, platform leaders create a supportive work environment through measures such as empowerment and communicating the corporate vision. This environment influences the cognition of organizational members, allowing employees to receive emotional and self—esteem support. Guo and Du (2013) pointed out that coworker support can help employees cope with stressful events. As a result, employees recognize their own importance and are motivated to innovate (Deng et al., 2020). Meanwhile, in a supportive environment, colleagues’ suggestions, knowledge sharing, etc. can enrich employees’ knowledge and skills, enabling them to meet the requirements of innovation tasks and increasing the likelihood of innovative behaviors (Rhoades and Eisenberger, 2002). Thus, the following hypothesis is proposed:

*H12*: Coworker support mediates the relationship between platform leadership and employee innovative behavior.

### 2.5 The mediating role of psychological empowerment between platform leadership and employee innovative behavior

Psychological empowerment focuses on employees’ internal perceptions and represents an intrinsic motivation process (Menon, 2001). Spreitzer (1995) posited that psychological empowerment refers to the extent to which employees perceive the delegation of authority from their leaders. According to the AMO theory, the relationship between platform leadership and psychological empowerment is one between the external opportunity environment and the internal motivational cognition. The positive impact of platform leadership is manifested in employees’ perception level of psychological empowerment. Platform leadership empowers employees, thereby fulfilling their need for work autonomy. Leaders’ equal and inclusive treatment of employees satisfies their need for a sense of belonging. Moreover, platform leadership, which emphasizes interactive collaboration, provides support to employees, meeting their need for competence. Platform leadership influences employees’ proactive innovation behavior by affecting their creative self—efficacy, which is also one of the dimensions of psychological empowerment. Xie (2022) found that platform leadership affects employees’ voice behavior by influencing their level of psychological empowerment. Therefore, this study argues that platform leadership can effectively enhance employees’ perception of psychological empowerment.

*H5*: Platform leadership exerts a significant positive influence on psychological empowerment.

According to the AMO theory, psychological empowerment promotes employees’ innovative behavior in two aspects. In terms of ability, psychological empowerment enhances employees’ self—efficacy. Individuals control their own behaviors through subjective beliefs and self—efficacy, acquire and utilize knowledge, and regulate their self—behaviors (Bandura, 1986). Employees with a high level of perceived psychological empowerment are energetic and show a more positive work attitude. They are confident in facing challenges, expanding knowledge and skills, which provides support for innovation (Yang Q. et al., 2022). In terms of motivation, psychological empowerment awakens employees’ intrinsic motivation. The consistent goals make employees love their work more due to its meaning and autonomy. The personal goals are highly consistent with the organization’s innovation goals. Additionally, the increase in employees’ participation in decision—making and access to resources facilitates knowledge integration and innovation. Under the mutual promotion of psychological perception and behavior, innovative ideas are transformed into actions (Bandura, 1986). Relevant studies have pointed out that psychological empowerment has a certain impact on employees’ work performance and proactive innovative work behavior, and it plays a positive role (Shi and Yang, 2015; Zhao et al., 2021). Employees with a higher level of perceived psychological empowerment at work are more likely to show proactive innovative behaviors (Chen, 2015; Yang Q. et al., 2022). Thus, the following hypothesis is proposed:

*H9*: Psychological empowerment significantly and positively impacts employee innovative behavior.

Platform leadership adheres to the concept of mutual fulfillment, emphasizing common growth and joint decision—making. It is more tolerant of employees, triggering positive psychological and behavioral factors. It supports employees in cognition, emotion, and morality, and grants sufficient work autonomy and decision—making power, strengthening employees’ perceived empowerment (Hao et al., 2021). Keller et al. (1995) found that trusting employees’ abilities and caring about and supporting their ideas and behaviors contribute to enhancing employees’ perceived empowerment. Thus, platform leadership helps improve employees’ psychological empowerment perception. Thomas and Velthouse (1990) and Spreitzer (1995) found that psychological empowerment is highly correlated with employees’ resilience and work autonomy. Psychological empowerment can mobilize employees’ innovation enthusiasm, enabling them to engage in risky work and stimulate creativity. Spreitzer (1995) argued that employees with high psychological empowerment can receive more leadership support. When the level of psychological empowerment is high, employees will generate more positive emotions at work, strengthen their intrinsic motivation, and be more willing to engage in creative work. Combining Hypothesis 4 and Hypothesis 8, this study argues that platform leadership will enhance subordinates’ psychological empowerment perception, which in turn will stimulate employees’ innovative behavior. Therefore, the following hypothesis is proposed:

*H13*: Psychological empowerment acts as a mediating factor in the relationship between platform leadership and employee innovative behavior.

**FIGURE 1:**
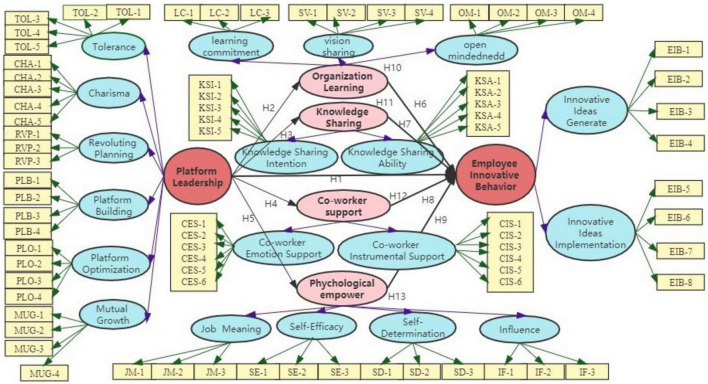
Research framework.

Based on Vroom’s (1964) AMO performance model and previous research, it is posited that employees’ effort levels determine their job performance, and the extent of task completion effort is influenced by their abilities and motivation. This study views employee innovative behavior as a complex system and incorporates it as a capability factor, psychological empowerment as a motivational factor, and platform leadership, organizational learning, and coworker support as opportunity factors. By examining the interplay among individuals, contexts, and opportunities, this study seeks to elucidate the impact mechanism of platform leadership, organizational learning, knowledge sharing, coworker support, and psychological empowerment on employee innovative behavior, within the framework of the AMO theory model. This article proposes the following framework as Figure 1.

The theoretical framework includes 13 main hypothesis as Table 1. Hypothesis H1 tests the relationship between platform leadership and employee innovation behavior, H2, H3, H4, H5 test the relationship between platform leadership on organizational learning, knowledge sharing, coworker support and psychological empowerment. H6, H7, H8, H9 examine whether organizational learning, knowledge sharing, coworker support and psychological empowerment have an impact on employee innovation behavior. Finally, we examine whether organizational learning, knowledge sharing, coworker support, and psychological empowerment (H10, H11, H12, H13) play a mediating role between platform leadership and employee innovative behavior.

**TABLE 1:** Hypothesis list.

Hypothesis no.	Contents of hypothesis
H1	Platform leaders have a positive and significant influence on employee innovative behavior
H2	Platform leaders have a significant positive impact on organization learning
H3	Platform leaders have a significant positive impact on Knowledge sharing
H4	Platform leaders have a significant positive impact on the coworker support
H5	Platform leaders have a significant positive impact on the employee’s psychological empower
H6	Organization learning have a significant positive impact on employee innovative behavior
H7	knowledge sharing have a significant positive impact on employee innovative behavior
H8	Coworker support have a significant positive impact on employee innovative behavior
H9	Psychological empowerment have a significant positive impact on employee innovative behavior
H10	Organization learning mediate the relationship between Platform leaders and employee innovative behavior. (mediator)
H11	Knowledge sharing mediate the relationship between Platform leaders and employee innovative behavior. (mediator)
H12	Coworker support mediate the relationship between Platform leaders and employee innovative behavior. (mediator)
H13	Psychological empower mediate the relationship between Platform leaders and employee innovative behavior. (mediator)

## 3 Research methodology

### 3.1 Research design

In alignment with the sequential explanatory design approach, this study initially employed quantitative methods to validate the proposed model and its 13 hypotheses, utilizing SPSS and Smart-PLS as analytical tools. Data collection was conducted in two phases: a pilot study followed by a formal study. The findings from the quantitative analysis were then used to inform the themes for semi-structured interviews during the qualitative analysis phase, aiming to uncover the factors and underlying mechanisms through which platform leadership influences employee innovative behavior. Finally, the study integrated and compared the quantitative and qualitative results, leading to the formulation of relevant discussions and research contributions.

The sample data for this research were gathered from Guangxi and Shanxi, regions ranked 19th and 20th in China’s economic hierarchy, as well as neighboring provinces. The study targeted employees in industries closely associated with digitization, networking, or intelligence, including manufacturing, software and information services, internet-based companies, and enterprises developing digital platforms, as the primary subjects for investigation.

### 3.2 Study 1: quantitative research, data analysis and hypothesis testing

#### 3.2.1 Sampling and instrument

To determine the sample size, the method by Wanichbancha (2006) was used, targeting 384 participants for reliable data at a 95% confidence level. Established scales were adapted for the Chinese context and validated through preliminary surveys and interviews. A Likert 5-point scale was used. Platform leadership included 6 dimensions and 25 items (Cronbach’s α = 0.947),with “tolerance,” “charisma,” “revolution planning,” “platform building,” “platform optimization,” and “mutual growth” as key dimensions (Hao et al., 2021). Employee innovative behavior comprised 2 dimensions and 8 items (Cronbach’s α = 0.829) (Scott and Bruce, 1994; Pamela et al., 1999; Gao, 2015). Organizational learning had 3 dimensions and 12 items (Cronbach’s α = 0.884) (Sinkula et al., 1997; Lv, 2013). Knowledge sharing consisted of 2 dimensions and 12 items (Cronbach’s α = 0.768–0.833) (Tian, 2015; Schepers et al., 2019). Coworker support included 2 dimensions and 12 items (Cronbach’s α = 0.925) (Liang and Fang, 2022; Tews et al., 2013). Psychological empowerment was also measured (Li et al., 2006; Spreitzer, 1995).

#### 3.2.2 Data collection

To validate the research framework, a pilot study was conducted before the formal survey. Based on feedback from colleagues and friends, 60 managers and employees from intelligent manufacturing and online platform companies in Shanxi and Guangxi were invited to complete an online questionnaire, yielding 56 valid responses. After analyzing the pilot data, the researchers used Corrected Item-Total Correlation (CITC) and construct reliability to optimize the scales. Items TOL-2, PLB-2, and PLO-4 in platform leadership were removed due to CITC values below 0.5 (0.458, 0.447, 0.475). This improved the α coefficients to 0.868, 0.889, and 0.909, enhancing scale reliability.

In the formal survey (Study 1), data were collected using a Chinese online questionnaire platform. Of the 550 collected questionnaires, 32 were excluded for response times under 100 s, leaving 518 valid responses, with an effective response rate of 94.2%. Sample characteristics include: 71% from Shanxi, 64% male, 83% aged 26–45, 80% with undergraduate education, 81% as grassroots supervisors and employees, and a majority with 0–5 years of work experience.

#### 3.2.3 Results and findings

##### 3.2.3.1 Test of common method bias

This study employs two methods to test for common method variance (CMV) bias. First, the Harman’s one—factor test was conducted using SPSS.21. The results showed that the variance explained by the first factor was only 38.772%, which is below the 40% criterion (Harman, 1961). Therefore, it indicates that there is no issue of common method bias. Second, referring to the common method factor analysis proposed by Liang et al. (2007), the ratio of the average squared loading of the substantive factors to the average squared loading of the common method variance (CMV) factor was used to determine the presence of common method bias. A larger ratio indicates a smaller problem of common method bias, as shown in Table 7. In this study, the final ratio is 18.7:10, which is relatively large, suggesting that there is no common method bias (Liang et al., 2007).

##### 3.2.3.2 Test results of reliability and validity

Tables 2, 3 report reliability test results: Cronbach’s Alpha (0.711–0.944) and composite reliability (CR) (0.795–0.954) exceed 0.7, indicating good reliability (Fornell and Larcker, 1981). All factor loadings surpass 0.6 (p < 0.001), and AVE values exceed 0.5, confirming convergent validity (Hair et al., 2011). Discriminant validity is supported as the square root of each AVE exceeds inter-variable correlations (Tables 4, 5), most HTMT values are below 0.85 (< 0.9) (Henseler et al., 2015), and items load more strongly on their respective variables (Hair et al., 2011).

**TABLE 2:** The reliability and validity test of the first order constructs.

First order constructs	Items	Loadings	Cronbach’s alpha	CR	AVE
TOL	TOL-1	0.791	0.830	0.886	0.662
	TOL-3	0.839			
	TOL-4	0.768			
	TOL-5	0.853			
CHA	CHA-1	0.89	0.933	0.949	0.790
	CHA-2	0.866			
	CHA-3	0.87			
	CHA-4	0.908			
	CHA-5	0.909			
PLB	PLB-1	0.855	0.835	0.901	0.752
	PLB-3	0.898			
	PLB-4	0.849			
RVP	RVP-1	0.936	0.929	0.954	0.875
	RVP-2	0.931			
	RVP-3	0.939			
PLO	PLO-1	0.914	0.917	0.947	0.857
	PLO-2	0.935			
	PLO-3	0.93			
MUG	MUG-1	0.93	0.944	0.960	0.857
	MUG-2	0.917			
	MUG-3	0.922			
	MUG-4	0.933			
CL	CL-1	0.7	0.825	0.884	0.656
	CL-2	0.859			
	CL-3	0.84			
	CL-4	0.842			
SV	SV-1	0.776	0.826	0.829	0.742
	SV-2	0.849			
	SV-3	0.905			
	SV-4	0.828			
OM	OM-1	0.81	0.830	0.887	0.664
	OM-2	0.854			
	OM-3	0.757			
	OM-4	0.835			
KSA	KSA-1	0.834	0.854	0.895	0.632
	KSA-2	0.77			
	KSA-3	0.741			
	KSA-4	0.776			
	KSA-5	0.848			
KSI	KSI-1	0.871	0.919	0.939	0.755
	KSI-2	0.822			
	KSI-3	0.902			
	KSI-4	0.889			
	KSI-5	0.86			
CES	CES-1	0.747	0.879	0.908	0.624
	CES-2	0.812			
	CES-3	0.767			
	CES-4	0.824			
	CES-5	0.751			
	CES-6	0.835			
CIS	CIS-1	0.819	0.818	0.863	0.525
	CIS-2	0.804			
	CIS-3	0.766			
	CIS-4	0.652			
	CIS-5	0.641			
	CIS-6	0.639			
WM	WM-1	0.919	0.877	0.924	0.804
	WM-2	0.927			
	WM-3	0.842			
SE	SE-1	0.914	0.898	0.937	0.831
	SE-2	0.942			
	SE-3	0.877			
SD	SD-1	0.918	0.920	0.949	0.862
	SD-2	0.933			
	SD-3	0.934			
IF	IF-1	0.88	0.862	0.915	0.783
	IF-2	0.885			
	IF-3	0.89			
EIBG	EIBG-1	0.823	0.917	0.942	0.802
	EIBG-2	0.926			
	EIBG-3	0.892			
	EIBG-4	0.937			
EIBI	EIBI-1	0.871	0.875	0.942	0.724
	EIBI-2	0.866			
	EIBI-3	0.877			
	EIBI-4	0.793			

**TABLE 3:** The reliability and validity test of the second order constructs.

Second order constructs	Items	Loadings	Cronbach’s alpha	CR (rho_a)	AVE
PL	TOL	0.797	0.921	0.938	0.718
	CHA	0.931			
	RVP	0.811			
	PLB	0.88			
	PLO	0.757			
	MUG	0.889			
OL	CL	0.863	0.88	0.926	0.807
	SV	0.881			
	OM	0.910			
KS	KSI	0.944	0.85	0.93	0.87
	KSA	0.917			
CS	CIS	0.927	0.867	0.938	0.883
	CES	0.950			
PE	WM	0.942	0.711	0.795	0.629
	SE	0.874			
	SD	0.887			
	IF	−0.287			
EIB	EIBG	0.898	0.761	0.853	0.744
	EIBI	0.822			

**TABLE 4:** The square root of the AVE value of variables and the correlation coefficient.

	CES	CHA	CIS	CL	CS	EIB	EIBG	EIBI	IF	KS	KSA	KSI	MUG	OL	OM	PE	PL	PLB	PLO	RVP	SD	SE	SV	TOL	WM
CES	**0.790**																								
CHA	0.511	**0.889**																							
CIS	0.763	0.548	**0.724**																						
CL	0.517	0.498	0.453	**0.813**																					
CS	0.950	0.565	0.927	0.521	**0.712**																				
EIB	0.532	0.487	0.482	0.678	0.543	**0.754**																			
EIBG	0.480	0.488	0.464	0.644	0.504	0.898	**0.895**																		
EIBI	0.429	0.333	0.352	0.510	0.421	0.822	0.489	**0.852**																	
IF	−0.353	−0.180	−0.471	−0.131	−0.428	−0.179	−0.185	−0.114	**0.885**																
KS	0.637	0.534	0.548	0.718	0.635	0.726	0.705	0.525	−0.226	**0.775**															
KSA	0.626	0.491	0.508	0.670	0.609	0.679	0.659	0.494	−0.262	0.917	**0.795**														
KSI	0.565	0.501	0.511	0.668	0.577	0.673	0.655	0.484	−0.169	0.944	0.735	**0.869**													
MUG	0.470	0.779	0.499	0.438	0.518	0.447	0.433	0.327	−0.167	0.483	0.442	0.455	**0.926**												
OL	0.645	0.632	0.633	0.863	0.683	0.770	0.742	0.564	−0.213	0.832	0.773	0.775	0.592	**0.735**											
OM	0.592	0.499	0.577	0.678	0.623	0.707	0.694	0.502	−0.227	0.781	0.727	0.727	0.500	0.910	**0.815**										
PE	0.553	0.627	0.618	0.484	0.623	0.555	0.526	0.418	−0.287	0.598	0.571	0.544	0.580	0.648	0.582	**0.723**									
PL	0.515	0.931	0.551	0.500	0.569	0.503	0.494	0.358	−0.181	0.540	0.490	0.513	0.889	0.654	0.532	0.642	**0.761**								
PLB	0.480	0.782	0.501	0.430	0.524	0.446	0.419	0.341	−0.150	0.475	0.445	0.440	0.746	0.591	0.488	0.555	0.880	**0.867**							
PLO	0.338	0.574	0.378	0.372	0.382	0.332	0.322	0.240	−0.066	0.371	0.288	0.391	0.629	0.471	0.377	0.415	0.757	0.752	**0.926**						
RVP	0.396	0.720	0.439	0.392	0.444	0.401	0.388	0.290	−0.160	0.416	0.385	0.388	0.657	0.524	0.428	0.511	0.811	0.648	0.557	**0.935**					
SD	0.431	0.531	0.496	0.369	0.494	0.435	0.409	0.331	−0.142	0.500	0.451	0.477	0.490	0.520	0.475	0.887	0.534	0.436	0.338	0.412	**0.928**				
SE	0.479	0.570	0.521	0.462	0.532	0.531	0.514	0.389	−0.169	0.544	0.529	0.488	0.530	0.598	0.528	0.874	0.596	0.539	0.386	0.486	0.626	**0.912**			
SV	0.591	0.651	0.632	0.633	0.652	0.643	0.616	0.475	−0.200	0.711	0.649	0.673	0.609	0.881	0.737	0.624	0.671	0.628	0.485	0.531	0.509	0.561	**0.862**		
TOL	0.379	0.771	0.393	0.376	0.412	0.418	0.427	0.276	−0.174	0.427	0.393	0.402	0.633	0.477	0.385	0.532	0.797	0.587	0.476	0.572	0.460	0.492	0.471	**0.814**	
WM	0.526	0.594	0.564	0.478	0.581	0.526	0.488	0.411	−0.186	0.555	0.533	0.503	0.546	0.625	0.552	0.942	0.606	0.528	0.414	0.478	0.804	0.766	0.609	0.480	**0.897**

The bold numbers represent the square roots of the average variance extracted (AVE) values for each variable, while the non - bold numbers are the correlation coefficients between this variable and other variables.

**TABLE 5:** Discriminant validity—Heterotrait-monotrait ratio (HTMT).

	CES	CHA	CIS	CL	EIBG	EIBI	IF	KSA	KSI	MUG	OM	PLB	PLO	RVP	SD	SE	SV	TOL	WM
CES																			
CHA	0.561																		
CIS	0.888	0.602																	
CL	0.603	0.565	0.536																
EIBG	0.532	0.526	0.532	0.737															
EIBI	0.48	0.362	0.395	0.593	0.537														
IF	0.405	0.198	0.616	0.154	0.206	0.124													
KSA	0.711	0.544	0.593	0.79	0.735	0.558	0.301												
KSI	0.629	0.542	0.576	0.767	0.713	0.533	0.189	0.819											
MUG	0.514	0.829	0.542	0.494	0.464	0.354	0.181	0.487	0.489										
OM	0.692	0.567	0.689	0.817	0.794	0.578	0.27	0.854	0.833	0.565									
PLB	0.557	0.884	0.582	0.515	0.474	0.392	0.172	0.525	0.502	0.84	0.585								
PLO	0.374	0.62	0.415	0.426	0.347	0.262	0.071	0.322	0.427	0.675	0.432	0.856							
RVP	0.437	0.772	0.487	0.446	0.419	0.315	0.175	0.429	0.421	0.701	0.487	0.735	0.602						
SD	0.479	0.573	0.545	0.422	0.447	0.363	0.155	0.506	0.519	0.525	0.544	0.497	0.367	0.446					
SE	0.538	0.623	0.592	0.535	0.565	0.437	0.191	0.599	0.539	0.576	0.611	0.623	0.425	0.532	0.689				
SV	0.693	0.744	0.747	0.766	0.705	0.55	0.238	0.766	0.774	0.69	0.891	0.757	0.557	0.607	0.586	0.654			
TOL	0.439	0.872	0.46	0.454	0.487	0.32	0.201	0.458	0.458	0.713	0.463	0.702	0.544	0.649	0.522	0.572	0.573		
WM	0.596	0.656	0.644	0.559	0.544	0.46	0.211	0.61	0.562	0.599	0.646	0.616	0.461	0.529	0.898	0.861	0.715	0.561	

##### 3.2.3.3 Multicollinearity test

In this study, the VIF values of each latent variable are all below Table 6. The data indicate that there is no severe multicollinearity problem among these variables.

**TABLE 6:** Collinearity of the latent variable indicators.

ITEMs	DV-EIB
IV-PL	2.326
M1-OL	2.812
M2-KS	2.756
M3-CS	2.048
M4-PE	2.507

##### 3.2.3.4 Structural equation model evaluation

To measure the goodness—of—fit and predictive ability of the model, according to the model prediction method proposed by Shmueli et al. (2019), Tables 7, 8 are obtained. All the errors of the PLS model were lower than those of the LM model. Thus, we can conclude that our model has strong predictive power. Combining the model evaluation index SRMR (Standardized Root Mean Square Residual) values of 0.078 and 0.081, the model fit is relatively good.

**TABLE 7:** Standardized root mean square residual (SRMR).

	Saturated model	Estimated model
SRMR	0.078	0.081

**TABLE 8:** PLS-predict.

Variable	Q^2^ predict	PLS-SEM_RMSE	LM_RMSE	PLS-LM
PL	0.592	0.425	0.605	−0.180
OL	0.224	0.590	0.639	−0.049
KS	0.268	0.587	0.588	−0.001
CS	0.357	0.678	0.679	−0.001
PE	0.312	0.776	0.781	−0.005
EIB	0.276	0.777	0.780	−0.003

##### 3.2.4.4 Hypothesis testing

**FIGURE 2:**
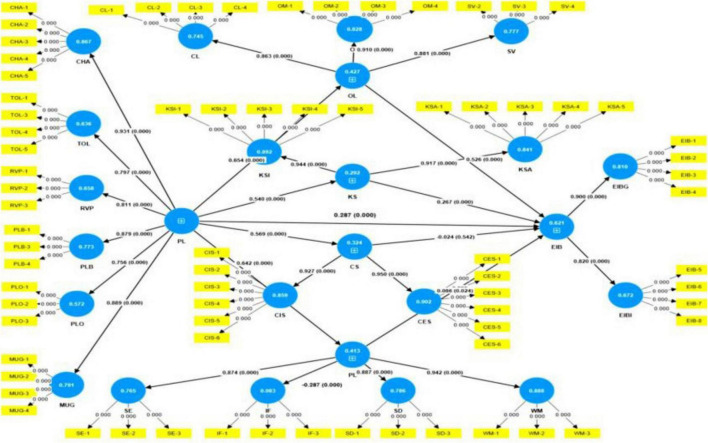
Results of the structural equation model (SEM) using Smart-PLS software.

Following Hair et al. (2019), researcher used a 5,000-sample bootstrap to report path coefficients, standard errors, *t*-values, and *p*-values for the structural model (Table 9 and Figure 2). Table 9 summarizes the hypothesis testing criteria. Platform Leadership exerts significant and positive direct effects on all variables except coworker support when impacting employee innovative behavior. Its strongest effects are on organizational learning, psychological empowerment and coworker support. Organizational Learning and Knowledge Sharing both have strong positive direct effects on employee innovative behavior. Coworker Support does not have a meaningful direct effect on employee innovative behavior, as indicated by the non-significant result. Psychological Empowerment has a positive but relatively weaker direct effect on employee innovative behavior, though still statistically significant. These findings highlight the central role of platform leadership in driving various organizational outcomes and suggest that fostering platform leadership could be a key strategy for enhancing innovation and organizational effectiveness.

**TABLE 9:** Hypothesis testing direct effects.

Hypothesis	Relationship	Original sample	Standard deviation	T statistics	*P*-values	Result
H1	PL - > EIB	0.287	0.071	4.024	0.000	Strongly supported
H2	PL - > OL	0.654	0.033	20.064	0.000	Strongly supported
H3	PL - > KS	0.540	0.038	14.074	0.000	Strongly supported
H4	PL - > CS	0.569	0.033	17.304	0.000	Strongly supported
H5	PL - > PE	0.642	0.029	22.379	0.000	Strongly supported
H6	OL - > EIB	0.526	0.060	8.733	0.000	Strongly supported
H7	KS - > EIB	0.267	0.055	4.815	0.000	Strongly supported
H8	CS - > EIB	−0.024	0.040	0.608	0.543	Rejected
H9	PE - > EIB	0.086	0.038	2.229	0.026	Weaker supported

##### 3.2.4.5 Mediation effect test

To test mediation hypotheses, we used bootstrap confidence intervals (Preacher and Hayes, 2004, 2008). The findings (Table 10) suggest that Platform leadership significantly influences Employee innovative behavior through organizational learning and knowledge sharing. Leaders who foster a culture of learning and knowledge sharing within the organizations are more likely to enhance employee innovation. However, Coworker support does not mediate this relationship, indicating that simply boosting an individual’s creative confidence may not directly translate into more innovative behavior. Additionally, psychological empowerment plays a role, though it has a smaller effect compared to OL and KS.

**TABLE 10:** Hypothesis testing indirect effects.

Hypothesis	Relationship	Indirect effect-β	Standard deviation	T statistics	*P*-values	Result
H10	PL - > OL - > EIB	0.344	0.043	7.950	0.000	Strongly supported
H11	PL - > KS - > EIB	0.144	0.031	4.680	0.000	Strongly supported
H12	PL - > CS - > EIB	−0.014	0.023	0.602	0.547	Rejected
H13	PL - > PE - > EIB	0.055	0.025	2.221	0.026	Weak supported

### 3.3 Study 2

#### 3.3.1 Research method and population

For Study 2, semi-structured interviews were conducted with 24 participants, following Liu (2020). Using Python and Word Cloud, analyses were performed on word frequency, semantic networks, and sentiment. The interviews, lasting 709 min, produced transcripts of 32,054 Chinese characters, translated into 19,993 English words. The jieba module segmented the corpus, and the word cloud module’s stopword dictionary removed non-meaningful words such as punctuation and pronouns.

#### 3.3.2 Results and finding

##### 3.3.2.1 Word frequency analysis and word cloud analysis

**FIGURE 3:**
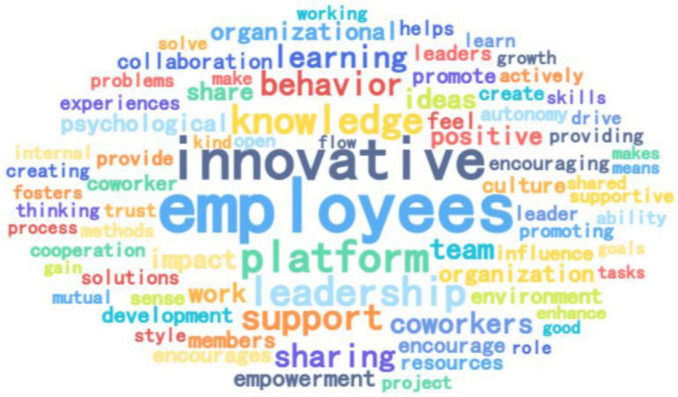
Word cloud of high-frequency words in interview texts.

Researcher focused on the top 30 high-frequency words, which are displayed in Table 11, due to space constraints. The word frequency analysis yielded a word cloud shown in Figure 3. Keywords such as “employees” (670), “platform” (309), “innovative” (498), and “leader” (283), as listed in Table 11, indicate respondents’ relevance to the topic. Knowledge, support, organization, sharing, and learning are central to the influencing mechanism of platform leadership and employee innovation behavior. The finding reveal that platform leadership fosters an innovative environment by empowering employees, encouraging idea sharing, and promoting collaboration. This supportive culture enhances knowledge sharing, learning, and psychological autonomy, leading to enhanced innovation behavior among employees.

**TABLE 11:** High-frequency words in interview texts.

No.	Words	Occurrences	No.	Words	Occurrences	No.	Words	Occurrences
1	Employees	670	11	Coworkers	152	21	Members	82
2	Platform	309	12	Team	151	22	Feel	76
3	Innovative	498	13	Ideas	136	23	Promote	72
4	Leadership	283	14	Encourage	135	24	Collaboration	71
5	Knowledge	270	15	Impact	132	25	Development	67
6	Support	246	16	Work	123	26	Environment	67
7	Behavior	198	17	Positive	115	27	Culture	64
8	Organizational	180	18	Share	95	28	Leaders	58
9	Sharing	182	19	Empowerment	94	29	Resources	58
10	Learning	175	20	Psychological	88	30	Autonomy	48

##### 3.3.2.2 Semantic network analysis

**FIGURE 4:**
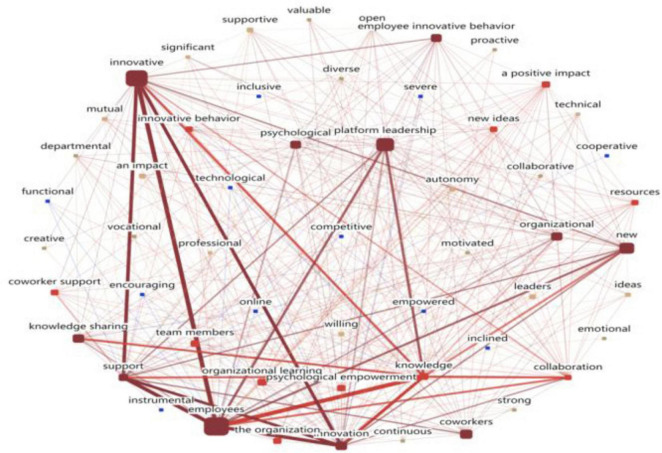
Semantic network relationship diagram.

Semantic network analysis examines relationships at the semantic level, revealing co-occurrence and clustering patterns. Figure 4 shows three core nodes: “employees,” “innovative,” and “platform leadership.” The “employees” cluster links platform, innovation, leadership, support, and knowledge, confirming people-oriented leadership. The “innovative” cluster connects employee, platform, share, idea, knowledge, and support, highlighting innovation’s reliance on employees, ideas, platforms, and knowledge sharin, it indicates that, within the mechanism through which platform leadership promotes employee innovation, respondents believe that sharing knowledge, providing a supportive platform and resources, and respecting and supporting employees’ ideas effectively facilitate the generation of innovative behavior. The “platform leadership” cluster ties platform, employee, encourage, knowledge, and support. The respondents indicated a stronger perception of platform leadership in its ability to provide resources, motivate employees, foster a knowledge-sharing culture, and offer growth opportunities. Notably, psychological empowerment, organization, innovation, learning, support, and knowledge sharing cluster around “employees,” indicating strong relationships.

##### 3.3.2.3 Sentiment analysis

**FIGURE 5:**
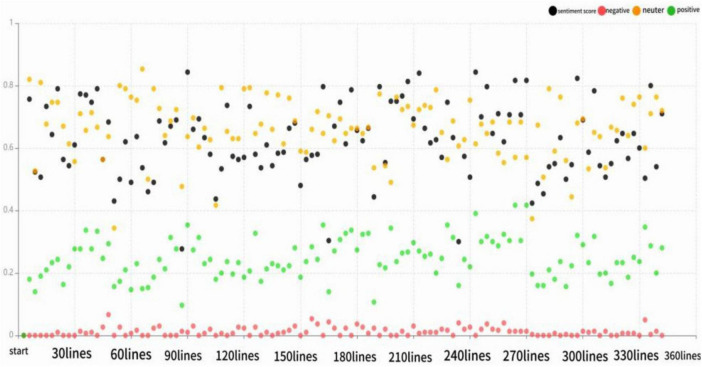
Distribution of *text sentiment scores.*

**FIGURE 6:**
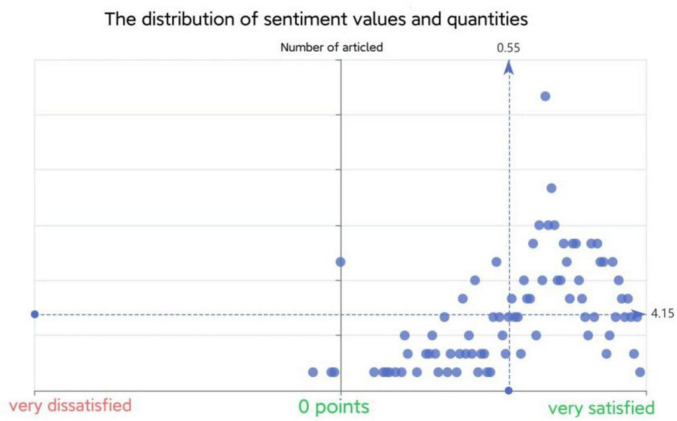
Distribution of sentiment values and quantities.

This study employed the open-source tool “Micro Word Cloud” for sentiment analysis of the corpus, classifying it into positive (> 0), neutral (= 0), and negative (< 0) categories based on an emotion dictionary. Results revealed 90.6% (925 sentences) were positive, 7.25% (74) neutral, and 2.15% (22) negative. This suggests interviewees generally view platform leadership’s impact on employee innovation positively, as detailed in Table 12 and Figures 5, 6. Data analysis indicated unanimous recognition of platform leadership’s positive influence on organizational learning, knowledge sharing and psychological empowerment, aligning with sentiment analysis findings. Moreover, 80% of the interviewees agreed that coworker support has a positive influence on employee innovative behavior.

**TABLE 12:** Basic information table for sentiment analysis.

Paragraph Sentiment classification	Number of positive paragraph	Number of negative paragraph	Number of neutral paragraph
Quantity	330	1	9
Sentence sentiment classification	Number of positive sentences	Number of negative sentences	Number of neutral sentences
Quantity	925	22	74

**FIGURE 7:**
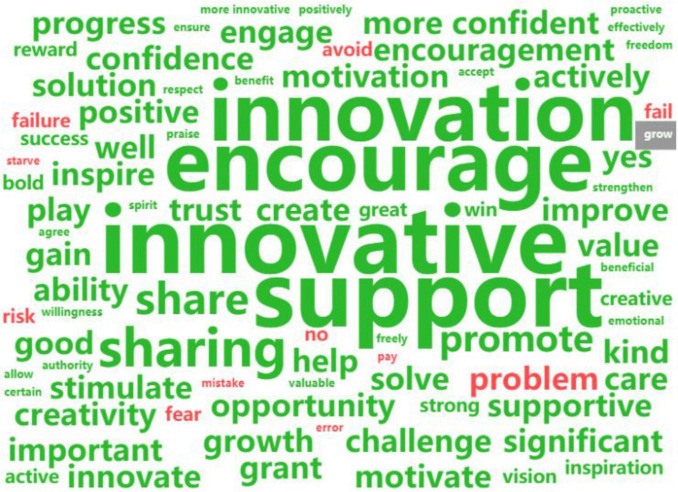
Word cloud of emotional word list.

Utilizing sentiment polarity classification, researcher generated a word cloud (Figure 7) featuring high-frequency words indicative of two emotional tones. Central to the internal mechanism linking platform leadership to innovative behavior are positive terms like “innovative,” “encourage,” “support,” “share,” “trust,” “improve,” “confident,” “growth,” and “motivation.” These elements foster employee innovation. Nevertheless, the presence of negative and neutral words is significant. Among 24 interviewees, 19 affirmed the positive influence of coworker support on innovation, while 4 expressed uncertainty and 1 saw no impact. Five interviews indicated no relevance between coworker support and innovation, highlighting high-frequency words such as “COVID-19,” “competitive,” and a Chinese proverb translating to “Teaching the disciple, starving the master.” The pandemic and remote work have decreased coworker intimacy, fostering competition over cooperation and withholding of knowledge. Automation and specialized labor divisions have also curtailed communication, leading some young employees to miss the supportive environment and its potential positive impact on innovation.

## 4 Conclusion and discussion

### 4.1 Conclusion

Employing a mixed-methods approach, this research integrates standardized quantitative analysis with in-depth qualitative exploration, yielding the following theoretical insights:

First, platform leadership significantly enhances employee innovation by leveraging its open and inclusive approach, resource and platform optimization, and collective growth mindset. This leadership style boosts intrinsic motivation and environmental opportunities, such as fostering open collaboration and knowledge sharing, which in turn elevates employees’ innovative behaviors.

Second, platform leadership positively impacts organizational learning, knowledge sharing, coworker support, and psychological empowerment. By providing a career platform and encouraging collective learning and knowledge sharing, platform leadership influences employees’ behaviors and attitudes.

Thirdly, organizational learning, knowledge sharing, and psychological empowerment significantly enhance employees’ innovative behaviors. These factors, including access to career platforms, organizational resources, coworker support, and employees’ psychological empowerment and personal beliefs, collectively influence innovation.

Fourthly, organizational learning, knowledge sharing, and psychological empowerment mediate the relationship between platform leadership and employee innovation, with organizational learning exerting the strongest influence. Platform leadership fosters a learning culture by establishing collaborative platforms, sharing resources, and supporting innovation. This culture enhances knowledge sharing, cross-departmental collaboration, and alignment between employees and leaders on organizational vision, thereby boosting employees’ innovative capabilities and behaviors. Furthermore, platform leadership empowers employees through trust and autonomy, reducing innovation-related fears and further stimulating innovative actions.

Lastly, coworker support has no significant impact on employees’ innovative behaviors. The mediating role of coworker support between platform leadership and employees’ innovative behaviors is not established. Platform leadership, with its inclusive attitude, appreciation of diversity, emphasis on collective progress, and provision of resources, information, and platforms for employees, as well as encouragement of knowledge sharing and open communication, positively influences support among colleagues. However, a small number of respondents believe that coworker support does not have a positive impact on innovative behaviors or that the impact is uncertain, and the underlying reasons for this require further research and verification.

### 4.2 Discussion

Firstly, platform leadership positively enhances employees’ innovative potential and behavior, aligning with findings from recent Chinese studies (Hao et al., 2021; Xu, 2022). This leadership style fosters innovation through inclusivity, charisma, strategic planning, platform development, optimization, and mutual growth, enabling employees to pursue achievement motivation and adapt to dynamic work environments. Grounded in social exchange theory, platform leadership empowers employees, emphasizing shared growth and organizational alignment. Employees reciprocate trust and support with pro-organizational behaviors, laying the groundwork for innovation. These findings enrich the understanding of platform leadership’s impact on employee innovation.

Secondly, organizational learning and knowledge sharing significantly promote employee innovation behaviors, with organizational learning having the greatest individual mediation effect, followed by knowledge sharing, and psychological empowerment having the least impact. According to social learning theory and AMO models, Personal beliefs and motivations are thought to have a greater influence on behavior than external factors. However, in this study, psychological empowerment showed a negative correlation in AVE values and related analyses, reducing its mediation effect. Therefore, the quantitative analysis indicates that organizational learning and knowledge sharing have larger effects than psychological empowerment. Qualitative interviews confirmed the positive relationships between these variables and platform leadership and employee innovation behaviors. The differences in mediation effects may be related to the timing of variable development and the characteristics of the study group, requiring further research for confirmation.

Thirdly, although platform leadership can significantly enhance organizational coworker support, the impact of coworker support on employee innovation behaviors requires further research, as does its mediating role between platform leadership and employee innovation. Qualitative interviews suggest three reasons for the weak mediating effect of coworker support: In the digital economy era, employees tend to seek work-related information through digital networks, thereby reducing the need for colleague support; most surveyed enterprises are closely integrated with platform-based economies and intelligent technologies. Clear divisions of labor among employees limit the need for information sharing and emotional support among colleagues. Additionally, the study was conducted post-COVID-19, during which isolation policies and remote online working arrangements further reduced colleague interactions, leading to a decline in coworker’s connectedness and a rise in individualism, thereby diminishing the effect of colleague support; The survey focused mainly on young and middle-aged employees, with 83% aged 26–45 and over 50% having 0–3 years of experience, This demographic often shoulders heavy workloads and long hours, which reduces opportunities for emotional interactions among colleagues, thereby affecting the impact of emotional support on innovation behavior.

### 4.3 Theoretical implications

This study adopts a sequential explanatory mixed-methods approach, combining quantitative structural equation modeling with qualitative content analysis of semi-structured interviews, utilizing techniques such as word frequency visualization, semantic network analysis, and sentiment analysis, based on a multi-perspective theoretical framework. It proposes a multiple mediation model more aligned with the complexities of real-world dynamic scenarios, not only validating the roles of these mediators but also elucidating their interactive effects, thereby expanding the theoretical research on platform leadership.

The study confirms the mediating roles of organizational learning and knowledge sharing, filling the gap in discussions about these influencing factors in platform leadership.

Furthermore, this study confirms the relationship between platform leadership and coworker support but also finds that the impact of coworker support on employee innovative behavior requires further verification, enriching the theoretical research on coworker support.

The study validates the mediating role of psychological empowerment, though its effect is not as strong as organizational learning and knowledge sharing, indicating that while internal motivational factors are crucial in motivating employee innovation, environmental opportunities and capabilities may play a larger role.

### 4.4 Managerial implications

Emphasize the selection and development of platform leaders to accelerate the construction of platform-oriented organizations where the organization, leadership, and employees can develop together. Focus on the mutual growth of leaders and employees, fully stimulate employees’ self-fulfillment needs, and achieve leadership self-fulfillment through the achievement of employees. Pay attention to cultivating organizational learning within the company, creating an inclusive and accepting learning atmosphere. Emphasize knowledge sharing by conducting regular training meetings, establishing internal knowledge sharing websites or platforms, and promoting personal development to enhance knowledge and emotional exchange among members within the organization. Prioritize employees’ perception of psychological empowerment feedback, strengthen the emotional connection between employees and the organization, support employee development and career planning, and inspire employee innovation behavior.

### 4.5 Limitations and future directions

Firstly, the study employed a one-time questionnaire, producing static cross-sectional data. Future research could adopt tracking surveys to capture temporal dynamics and enhance persuasiveness.

Secondly, the sample primarily came from Shanxi and Guangxi, limiting geographical diversity and affecting generalizability. Future studies can choose to include sample data from countries with different cultural backgrounds (such as Europe, America, Southeast Asia, Africa, etc.) to verify the cross-cultural applicability of platform leadership.

Lastly, self-reported data may introduce common method bias. Future studies should incorporate multi-source data for cross-validation and enhance conclusion credibility

Fourthly, the study is empirical and set within a Chinese context. While the scales for measuring platform leadership are based on Chinese research, their theoretical underpinnings stem from Western studies. This blend may constrain the cross-cultural applicability of the scales used. Future research could consider adapting or developing scales that are more culturally resonant across different societies.

Lastly, the relationship between platform leadership and employee innovation behavior might involve various mediating and moderating influences, including possible chain mediation effects. This study did not account for certain potential mediators such as organizational self-esteem, organizational culture, team psychological safety, and perceptions of insider status. Additionally, internal and external environmental factors were not factored into the analysis.

## Data Availability

The raw data supporting the conclusions of this article will be made available by the authors, without undue reservation.
